# Hepatocyte growth factor/c‐Met signaling axis in human diseases: Mechanistic insights and therapeutic potential

**DOI:** 10.1002/ccs3.70075

**Published:** 2026-05-10

**Authors:** Hongqin Sun, Hao Shu, Qian Zhang, Jishan Zheng, Yunyan Ma, Dengwang Chen, Jidong Zhang, Tao Song

**Affiliations:** ^1^ Department of Immunology Zunyi Medical University Zunyi China; ^2^ Special Key Laboratory of Gene Detection & Therapy of Guizhou Province Zunyi Medical University Zunyi China; ^3^ Guizhou Provincial Key Laboratory of Cancer Prevention and Treatment Zunyi Medical University Zunyi China

**Keywords:** c‐Met receptor, clinical translation, disease‐targeted therapy, hepatocyte growth factor (HGF), HGF/c‐Met pathway, signal transduction, tissue regeneration

## Abstract

Hepatocyte growth factor (HGF) is a multifunctional cytokine that activates the tyrosine kinase activity of its specific receptor, c‐Met (mesenchymal–epithelial transition factor). This activation subsequently regulates downstream signaling pathways such as PI3K/Akt and Ras/MEK, ultimately mediating key biological processes including epithelial cell migration, proliferation, morphogenesis, and damaged tissue regeneration. Accumulating evidence indicates that the HGF/c‐Met pathway is critical for embryonic development and tissue homeostasis. Dysregulation of this pathway—whether excessive activation or suppression—is closely associated with the pathogenesis of numerous human diseases. This review systematically synthesizes recent research data on the HGF/c‐Met pathway and elucidates its disease‐related mechanisms: it promotes malignant tumor progression, participates in viral infectious disease pathogenesis, facilitates tissue injury repair, and is implicated in diabetes mellitus and Alzheimer's disease. It further clarifies the pathway's fundamental biological functions, summarizes potential therapeutic strategies targeting this pathway (e.g., c‐Met inhibitors, HGF antagonists, and microRNA‐mediated regulation), and discusses challenges in clinical translation (e.g., poor target specificity and drug resistance), providing a theoretical reference for subsequent disease‐targeted therapy research.

## Graphical abstract

Figure 1 visually summarizes the core biological functions and therapeutic potential of the HGF/c‐Met signaling axis.

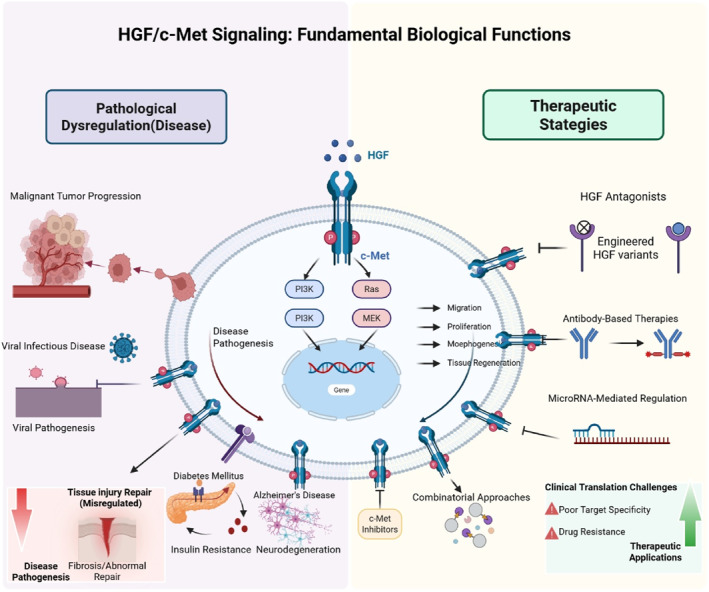

## 1 INTRODUCTION

Hepatocyte growth factor (HGF) comprises a large subunit (α subunit) and a small subunit (β subunit) and is an acid‐ and heat‐sensitive protein.^1^, ^2^ It plays a pivotal role in cell survival, tissue regeneration, and the attenuation of fibrosis and chronic inflammation.^3^, ^4^ Its specific receptor, c‐Met, consists of an α‐chain and a β‐chain linked by disulfide bonds and exhibits intrinsic tyrosine kinase activity,^5^ and c‐Met is recognized as an oncogene involved in cell migration and proliferation;^6^, ^7^ upon binding of HGF, the HGF/c‐Met pathway is activated via autocrine or paracrine mechanisms, leading to the downstream activation of multiple signaling cascades, including the Ras/MEK, p120/STAT3, Src/FAK, and PI3K/Akt pathways.^8^, ^9^, ^10^, ^11^ Under normal physiological conditions, the HGF/c‐Met pathway is crucial for embryonic maturation and development. In early embryonic stages, this pathway modulates the proliferation of embryonic stem cells; subsequently, it induces the epithelial–mesenchymal transition (EMT) by regulating the polarity and motility of differentiated cells.^12^, ^13^, ^14^ This EMT process not only preserves the differentiation potential of multipotent cells but also endows them with migratory capacity during organogenesis.^15^, ^16^, ^17^ Concurrently, the HGF/c‐Met pathway participates in tissue injury repair and regeneration. In multiple tissue injury models, activation of this pathway promotes repair, enhances cell survival, and mitigates chronic inflammation and fibrosis.^18^, ^19^

However, aberrant activation of the HGF/c‐Met pathway can also drive malignant progression. For instance, HGF/c‐Met signaling in vascular endothelial cells accelerates tumor angiogenesis and metastasis.^20^, ^21^ Moreover, hypoxia‐induced amplification of HGF signaling further enhances tumor proliferation.^22^ Consequently, the most critical biological effects induced by the HGF/c‐Met pathway encompass anti‐apoptosis, cell differentiation, migration, invasion, and angiogenesis, whilst simultaneously participating in both injury repair and tumorigenesis.^23^

**FIGURE 1:**
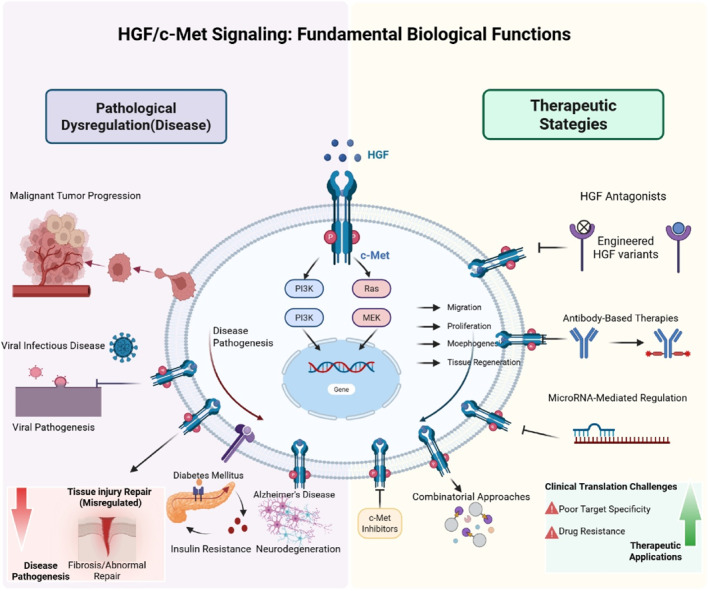
HGF/c‐Met signaling: fundamental biological functions. This schematic summarizes the HGF/c‐Met signaling axis: HGF activates c‐Met and downstream PI3K/Ras pathways, mediating cell migration, proliferation, morphogenesis, and tissue regeneration. Pathway dysregulation is closely linked to multiple human diseases, including malignant tumors, viral infections, disordered tissue repair, diabetes mellitus, and Alzheimer’s disease. This figure also outlines targeted therapeutic strategies (e.g., c‐Met inhibitors, HGF antagonists, antibody therapies, microRNA regulation) and key clinical translation challenges (poor target specificity, drug resistance), providing a clear visual reference for HGF/c‐Met‐targeted research. HGF, hepatocyte growth factor.

**FIGURE 2:**
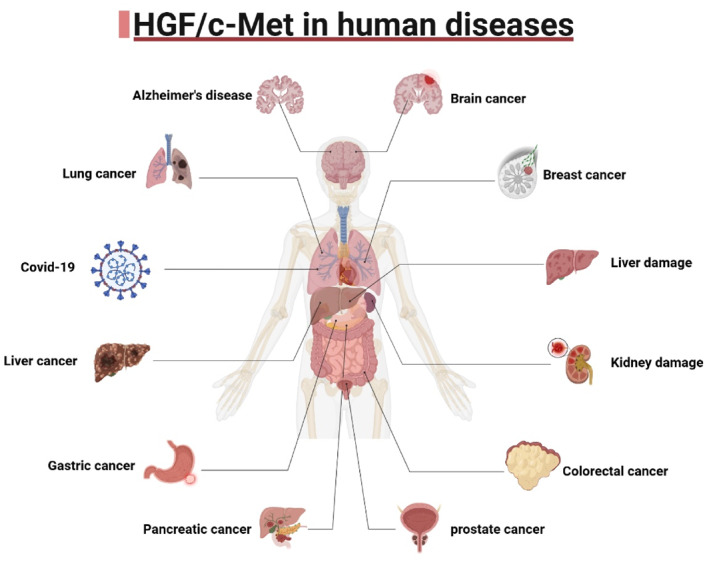
HGF/c‐Met in human disease. Targeted therapeutics primarily influence the biological effects of this signaling pathway and associated pathways by altering its expression to achieve disease modification, according to the research that is currently available on the HGF/c‐Met pathway. With differing degrees of success, pertinent targeted therapies have currently been examined in a range of tumor types, muscle injury, kidney injury, liver injury, Parkinson's disease. HGF, hepatocyte growth factor.

In this review, we synthesize recent evidence on the involvement of the HGF/c‐Met pathway in cancers, tissue injury, viral diseases, and neurodegenerative disorders. We also explore its fundamental functions and current therapeutic strategies targeting key components of this signaling axis. The overall framework and biological processes are illustrated in Figures 1 and 2, and the roles of the HGF/c‐Met signaling axis in various human diseases are summarized in Table 1.

**TABLE 1:** HGF/c‐Met in human diseases.

Diseases	Name	Targets	Mechanisms	References
Cancer	Lung cancer	DFX117	c‐Met could be targeted by DFX117 which can induce apoptosis of NSCLC	24
	Gastric cancer	PI3K/Akt, NF‐κB	Promoted HPA which could promote the shedding of associated cytokines in several types of tumors expression	25
	Liver cancer	EMT	c‐MET knockdown inhibits EMT, reducing HCC cell migration and invasion	
	Colorectal cancer	SU11274	HGF/c‐Met signaling pathway could be affected by SU11274 which is a c‐Met inhibitor. Then the different subgroups of colon cancer cells could be inhibited	26
	Breast cancer	EMT, CTX III	Involved the inactivation of the HGF/c‐Met‐mediated ERK1/2 and PI3K/Akt pathways in MDA‐MB‐231 cells	27
	Prostate cancer	PI3K/Akt	Which is the phosphorylation cascade downstream of HGF/c‐ met can regulate EMT. HGF‐induced EMT pathway	28
	Pancreatic cancer	NK4 MTOR/NGF	(1) Blocked the stromal–tumors interaction caused by HGF/c‐Met pathway and resist tumor angiogenesis (2) Promoted PNI in PC	29, 30, 31, 32
	Brain tumor	PI3K, STAT3 IRES	(1) Increased by the activation of HGF/c‐met pathway, resulting in increased tumor cell growth and angiogenesis (2) Mediated the translation of the coding region of the cross‐junction amino acid encoding of c‐Met	33, 34
	Cervical cancer	miR‐34a miR‐206	miR‐34a which acted as a tumor suppressor and miR‐206 inducing cell cycle arrest	35
	Bladder cancer	MAKP	Regulated HGF/c‐Met beneficial to tumor progression	36
Virus disease	Hepatitis C	CDH1	Loss of CDH1 expression was associated with upregulation of hepatocyte proliferation promoters MET and YAP1	37
	COVID‐19	HGF	HGF was a pleiotropic cytokine with anti‐inflammatory	38
	Pneumonia	TGF‐α/EGFR	Regulated the repair of damaged alveolar epithelium by stimulating cell migration and proliferation	39
Damage repair	Kidney injury	PPAR‐γ RA	Counteracted the antifibrotic effects of mesangial cells and promoted kidney injury repair	40, 41
	Liver injury	TGF‐β	Oxidative stress and apoptosis of hepatocytes are controlled	42
	Muscle injury	CAMKKβ AMPK	Promoted muscle regeneration to treat injured muscles	43
Other	Diabetes	NF‐κB	NF‐κB is related to apoptosis, and its activation is inhibited by HGF/c‐Met pathway	44

Abbreviations: AMPK, AMP‐activated protein; CAMKKβ, calcium/calmodulin‐dependent protein kinase beta; CDH1, E‐cadherin; CTX III, cardiotoxin III; EGFR, epidermal growth factor receptor; EMT, epithelial‐mesenchymal transition; HCC, hepatocellular carcinoma; HGF, hepatocyte growth factor; HPA, heparanase; NF‐κB, nuclear factor‐κB; NGF, nerve growth factor; NSCLC, non‐small cell lung cancer; PC pancreatic cancer; PNI, perineural invasion; PPAR‐γ, proliferator‐activated receptor gramma; TGF‐α, transforming growth factor α; YAP1, Yes‐associated protein 1.

## 2 HGF/c‐MET IN CANCERS

### 2.1 Lung cancer

**FIGURE 3:**
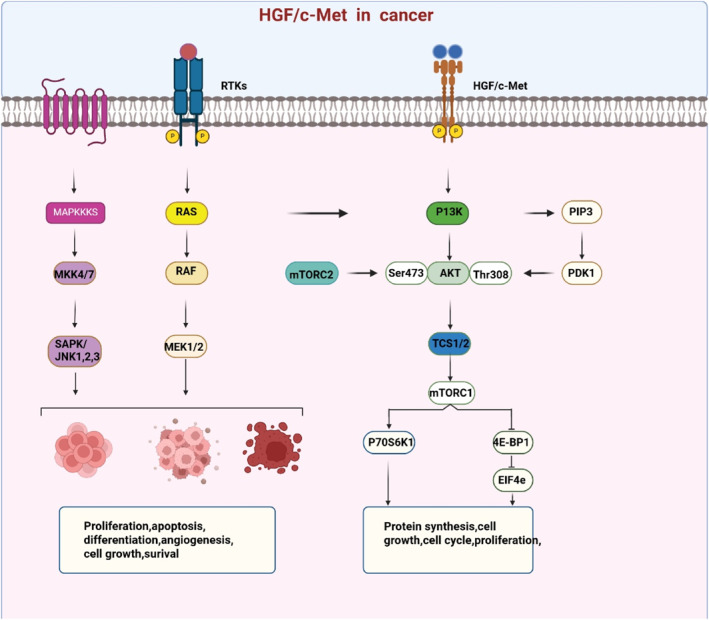
HGF/c‐Met in cancer. The HGF/c‐Met signaling pathway can activate PI3/Akt as well as PIP3 signaling transactivation to regulate the downstream Tcs1/2, mTORC1, and receive the regulation of RTKs and other pathways to inhibit tumor cell growth, cell metastasis, apoptosis, cell differentiation and other biological processes. HGF, hepatocyte growth factor.

The HGF/c‐Met pathway is frequently dysregulated in lung cancer, and patients exhibit elevated levels of HGF and c‐Met compared to healthy individuals.^24^, ^45^, ^46^ Thus, the HGF/c‐Met pathway may serve as a novel therapeutic target for lung cancer patients.^47^ A c‐Met antisense construct driven by the U6 snRNA promoter has been shown to downregulate c‐Met expression and promote apoptosis in lung cancer cells.^45^, ^46^ The c‐Met‐dependent heat shock protein 90 inhibitor geldanamycin can cause apoptosis in small cell lung cancer (SCLC) cells and decrease the growth and viability of some SCLC cell lines.^46^ DFX117, a dual inhibitor targeting both c‐Met and PI3Kα, exerts anti‐tumor effects in non‐small cell lung cancer (NSCLC) cells.^48^ Overexpression of microRNA‐200a (miR‐200a) reduces HGF expression, thereby suppressing migration and invasion while enhancing apoptosis and radiosensitivity in NSCLC cell lines.^47^ Notably, c‐Met overexpression is associated with poor prognosis in NSCLC, and c‐Met inhibitors have shown promising anti‐tumor activity in preclinical and clinical studies.^49^ The regulatory mechanism in cancer is further demonstrated in Figure 3.

### 2.2 Gastric cancer

The HGF/c‐Met pathway exerts a substantial impact on the proliferation, invasion, metastasis, and angiogenesis of gastric cancer. Patients with gastric cancer exhibit higher positivity rates for HGF and c‐Met compared to those with gastritis or gastric ulcers.^25^, ^49^, ^50^ NK4, a dual‐function antagonist that inhibits both HGF and angiogenesis, exerts promising therapeutic effects in preclinical models of gastric cancer.^51^ MiR‐16, which is significantly downregulated in gastric cancer tissues, suppresses tumor progression by directly inhibiting the HGF/c‐Met signaling pathway.^52^ Mechanistically, HGF/c‐Met activates PI3K/Akt/NF‐κB signaling, leading to upregulation of heparanase (HPA).^53^ In a dose‐dependent manner, monoclonal antibody 3936, which targets the human uPA urokinase plasminogen activator receptor, can inhibit MKN74 tumor cell invasion caused by HGF.^54^ Additionally, the long non‐coding RNA HOTAIR promotes EMT and metastasis via the HGF/c‐Met/snail axis.^55^ In conclusion, the HGF/c‐Met pathway can activate PI3K/Akt‐NF‐κB signaling and promote HPA expression and subsequent tumor metastasis, providing a strong experimental basis for targeted inhibition of gastric cancer occurrence and development.

### 2.3 Liver cancer

Hepatocellular carcinoma (HCC) is one of the most lethal malignancies worldwide. Activation of c‐Met signaling in carcinoma of the liver is associated with an invasive morphology and an unfavorable prognosis.^56^, ^57^ Abnormal c‐Met expression is associated with tumor recurrence and Metastasis because it stimulates cell proliferation, the EMT, and migration in HCC cells in HCC tissues.^26^ Conversely, c‐Met knockdown inhibits EMT, reducing HCC cell advancement and an assault.^58^ Circular RNA EIF3I (circEIF3I) is significantly upregulated in HCC tissues, and it can sponge miR‐526b‐5p to post‐transcriptionally regulate the downstream expression of HGF.^59^ Knockdown of circEIF3I decreases cell survival, EMT, migration, invasion, and angiogenesis in HCC cells while increasing cell apoptosis.^59^ Arf‐GAP with SH3 domain, ANK repeat and PH domain‐containing protein 2 (ASAP2) is a multifunctional protein that plays a key role in cell signaling,^60^ which enhances GTPase activity and activates receptor tyrosine kinases, and interacts with c‐Met to drive HCC invasion and recurrence.^61^ Investigating the interaction between ASAP2 and c‐Met can provide new insights into the prevention and inhibition of HCC, as well as the underlying mechanisms of HCC initiation. These findings suggest that c‐Met serves as a central node in HCC signaling networks.

### 2.4 Colorectal cancer

Colorectal cancer represents one of the most prevalent malignancies worldwide, yet it remains refractory to the majority of currently available chemotherapeutic agents.^62^ Increasing evidence has demonstrated that the HGF/c‐Met signaling pathway is involved in the formation of malignant tumor phenotypes, particularly playing a crucial role in the development of resistance to anticancer drugs.^63^ As a selective c‐Met inhibitor, SU11274 can effectively block HGF/c‐Met signaling and thereby inhibit the proliferation of colon cancer cells^62^; in addition, 5‐fluorouracil (5‐FU) can enhance the apoptotic effect in NK4‐expressing CT26 cells by inhibiting the intracellular activation of the HGF/c‐Met pathway^63^, whereas Lingqi capsule extract, a commonly used traditional Chinese medicine compound extract, can also significantly increase the apoptosis rate of colon cancer cells and induce corresponding changes in the protein levels and mRNA expression of HGF and c‐Met after treatment.^64^

### 2.5 Breast cancer

HGF/c‐Met signaling plays a critical role in driving the progression of breast cancer.^65^ Osteoblasts have been shown to promote bone metastasis of breast cancer via activating the HGF/c‐Met pathway.^66^ Amplification of the c‐Met receptor, also known as the mesenchymal–epithelial transition factor, is closely associated with chemotherapy failure in breast cancer patients.^28^ Cancer‐associated fibroblasts (CAFs) are key components of the tumor microenvironment (TME).^27^ CAFs secrete HGF, activating c‐Met signaling and promoting proliferation, EMT, and radioresistance in breast cancer cells.^27^ In breast tumors, the expression levels of HGF, c‐Met, HGFA (HGF activator), and HAI‐1/2 (HGF activator inhibitor 1/2) are markedly elevated in breast tumor tissues.^67^ Boswellia frereana extract (BFE) inhibits HGF‐ and c‐Met‐mediated phosphorylation and suppresses invasion.^65^ Inhibition of matriptase blocks the conversion of inactive pro‐HGF to mature active HGF and subsequent c‐Met‐mediated signaling, thereby effectively suppressing the invasion and proliferation of inflammatory breast cancer cells.^68^

Metastatic dissemination of breast cancer is initiated by EMT, a process that positively correlates with disease progression in patients.^69^, ^70^ Osthole, a natural coumarin derived from the traditional Chinese medicinal herb Cnidium monnieri, suppresses EMT in breast cancer cells by targeting the HGF/c‐Met–Akt/mTOR signaling axis.^69^ Cardiotoxin III (CTX III), a basic polypeptide isolated from the *Naja naja atra* venom, has been shown to exhibit anticancer activity.^71^ CTX III reverses EMT and inactivates HGF/c‐Met‐mediated ERK1/2 and PI3K/Akt signaling in MDA‐MB‐231 cells, consequently inhibiting HGF/c‐Met‐induced cell proliferation and invasion.^70^ Difluoromethylornithine intervention attenuates c‐Met phosphorylation and Matrigel invasion of MDA‐MB‐435 cells induced by HGF.^72^ Downregulation of suppressor of cytokine signaling 7 enables phospholipase C gamma 1 to inhibit HGF/c‐Met signaling during in vitro proliferation and migration of breast cancer cells.^73^

### 2.6 Prostate cancer

The HGF/c‐Met pathway is critically implicated in the progression of prostate cancer.^74^ Activation of c‐Met drives malignant phenotypes in prostate cancer and promotes the acquisition of cancer stem‐like properties.^74^ In men with prostate cancer, elevated HGF expression is associated with biological recurrence following radical prostatectomy, identifying HGF as a novel biomarker for prostate cancer stem cells/cancer‐initiating cells (CSCs/CICs).^29^ Downstream of HGF/c‐Met signaling, the PI3K/Akt phosphorylation cascade serves as a key regulator of EMT in prostate cancer. Diosgenin has been shown to target the HGF‐induced EMT pathway, representing a potential therapeutic strategy for prostate cancer.^69^ Furthermore, anti‐HGF rabbit monoclonal antibody (RabMAb) effectively blocks the HGF/c‐Met axis and its downstream activation, thereby representing a promising candidate for prostate cancer therapy.^75^ Given that androgen signaling represses c‐Met expression, combinatorial strategies integrating androgen deprivation therapy with HGF/c‐Met inhibition may yield superior therapeutic efficacy in the clinical management of prostate cancer.^30^

### 2.7 Pancreatic cancer

Arguably those most dangerous tumors and one that is challenging to treat is pancreatic cancer (PC).^76^, ^77^ Pancreatic stellate cells (PSCs) produce HGF, promoting tumor aggressiveness.^77^ PSCs and PC cells interact to accelerate the growth of PC, a phenomenon known as stromal‐tumor interaction,^78^, ^79^ and this interaction could potentially be controlled by the HGF/c‐Met pathway.^77^ Hypoxia is a critical component of the pancreatic TME and promotes tumor growth and dissemination.^80^ The HGF/c‐Met pathway‐mediated stromal–tumor interaction under hypoxia worsens the prognosis of patients.^81^ PSCs and cancer cells differ in this particular route. PSCs release the ligand HGF, and PC cells contain the receptor c‐Met.^31^ Therefore, one potential target for PC treatment is the suppression of matrix tumor contact mediated by the HGF/c‐Met pathway.^79^ HGF inhibitor, c‐Met inhibitor, and gemcitabine were utilized in Rucki and Xu investigation to stop PC metastasis and stop tumor growth.^76^, ^78^ This therapy can inhibit angiogenesis and lessen postoperative recurrence.^32^ However, long‐term use of HGF/c‐Met inhibitors may lead to resistance via upregulation of Sonic hedgehog (Shh) signaling.^75^ In addition to being an HGF antagonist, NK4 also inhibits angiogenesis, which means that it can prevent tumor angiogenesis by blocking the stromal‐tumor contact brought on by the HGF/c‐Met pathway. Consequently, it can be applied to PC as a targeted anti‐metastasis therapy.^82^ The circulating levels of c‐Met in PC and malignant symptoms of PC are linked to perineural invasion (PNI).^83^ According to Li Nan and Tao Qin's research, triggering the HGF/c‐Met pathway increases nerve growth factor (NGF) expression and cancer cell Metastasis. It also stimulates the mTOR/NGF axis in pancreatic tumors, which in turn promotes PNI in PC.^77^, ^83^

### 2.8 Brain tumor

The HGF/c‐Met pathway is critically involved in the development and progression of various human malignancies, including brain tumors.^33^ One type of brain tumor is a pituitary adenoma. Pituitary adenomas express HGF, c‐Met, PI3K, pAkt, and STAT3. The phosphorylation of the HGF/c‐Met pathway increases the production of the subsequent effectors PI3K/phosphor‐Akt and STAT3/phospho‐STAT3, which in consequently increases the proliferation of tumor cells and angiogenesis.^34^ In glioblastoma (GBM), the most common primary malignant brain tumor, HGF/c‐Met signaling drives tumor growth and progression, and activating c‐Met mutations contribute to the malignant transformation of low‐grade gliomas to secondary GBM,^84^ c‐Met knockdown suppresses cell proliferation, migration, and invasion, whereas c‐Met amplification enhances invasiveness, and a functional IRES has been identified in circ‐HGF that mediates HGF translation.^85^ In neuroblastoma, aberrant HGF/c‐Met signaling constitutively activates STAT3, PLC‐γ, and MAPK cascades to promote invasion, while inducing tPA and MMP‐2 to accelerate malignant progression.^86^ In medulloblastoma, HGF/c‐Met activation induces Akt and MAPK phosphorylation, regulates p27 and CDK2 to promote cell‐cycle progression, and protects tumor cells from chemotherapy‐induced apoptosis.^87^ Collectively, hyperactivation of the HGF/c‐Met pathway is closely associated with malignant progression across multiple CNS tumors, supporting its potential as a therapeutic target in combination with chemotherapy and radiotherapy.^86^, ^87^, ^88^ Emd1214063, a selective c‐Met inhibitor, has been shown to trigger neuroblastoma cell death and suppress tumor progression via inhibition of the HGF/c‐Met signaling axis and MEK phosphorylation.^88^ Accordingly, both shRNA silencing of c‐Met and treatment with the c‐Met inhibitor PF‐2341066 hold great promise as targeted therapeutic approaches for neuroblastoma.^33^, ^34^

### 2.9 HGF/c‐Met signaling in other human malignancies

Adult T‐cell leukemia/lymphoma (ATL) comprises two subtypes: Peripheral blood‐type ATL (PB‐ATL) and non‐PB‐ATL, with non‐PB‐ATL displaying greater malignancy and aberrant activation of the HGF/c‐Met pathway; bromodomain and extra‐terminal motif inhibitors exert anti‐tumor effects by targeting HGF production and regulating H3K27ac/BRD4 in this signaling axis, thereby suppressing non‐PB‐ATL growth.^36^, ^89^ In bladder cancer, the Crk adapter protein activates HGF/c‐Met signaling and its downstream Ras–ERK–MAPK cascade to drive tumor progression, and since both pathways are oncogenically activated, targeted Crk knockdown represents a promising therapeutic approach for this malignancy.^35^, ^90^ In cervical cancer, the progression and prognosis are closely linked to HGF/c‐Met signaling, which is accompanied by upregulated expression of BCL2 and c‐Met, alongside downregulated expression of Notch3 pathway targets, the tumor‐suppressive miR‐34a, and the cell cycle‐arresting miR‐206, suggesting that miR‐34a and miR‐206 may serve as effective therapeutic agents for cervical cancer.^37^, ^91^

## 3 HGF/c‐MET IN VIRUS DISEASE

### 3.1 Hepatitis C

**FIGURE 4:**
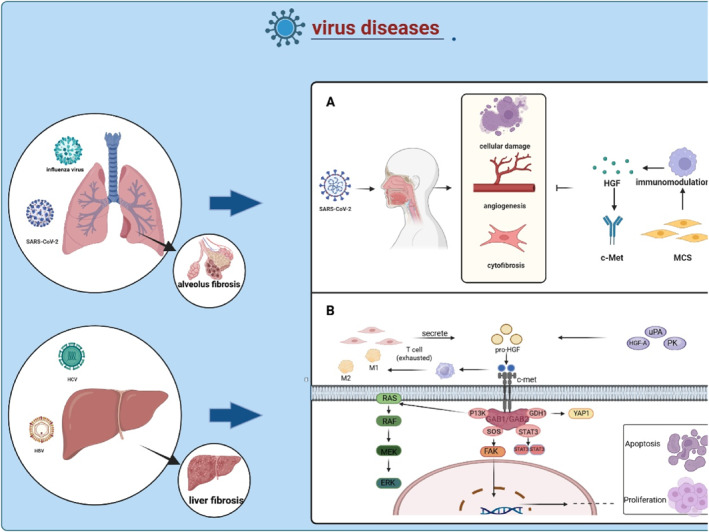
HGF/c‐Met in virus disease. (A) SARS‐CoV‐2 infected patients exhibit endothelial cell damage, neoangiogenesis, cellular fibrosis, etc., and MSCs can inhibit their biological processes by producing HGF in conjunction with receptor c‐Met binding. (B) HGF receives MSC‐stimulated secretion, binds to the receptor c‐Met, and works with the downstream PI3, SOS, and STAT3 pathways to regulate cell proliferation and apoptosis. HGF, hepatocyte growth factor; MSCs, mesenchymal stem cells.

Beyond malignant tumors, dysregulation of the HGF/c‐Met pathway also plays a pivotal role in the pathogenesis of viral infectious diseases by modulating viral replication, host immune response, and tissue damage repair.^92^, ^93^ Globally, the greatest widespread cause for ongoing hepatitis and liver disease is the hepatotropic virus known as the hepatitis C virus (HCV).^92^, ^94^ Alongside the suppression of hepatocyte and hepatic stem cell growth is a hallmark of HCV‐cirrhosis.^95^ Nevertheless, the c‐Met gene can act as a crucial hub since HGF and its receptor Met are necessary for the expansion and propagation of liver cancer, primarily depending on the reactivation of the receptor's tyrosine kinase.^38^ The upregulation of Met and yes‐associated protein 1 (YAP1), two promoters of hepatocyte proliferation, is linked to loss of E‐cadherin (CDH1) expression. By using Cox regression with tumor stage adjustment, CDH1, Met, and YAP1 were found to be distinct indicators of the absence of recurrence.^95^ High urine HGF amounts are linked to more fibrosis in patients with HCV infection, according to data on patients with single HCV infection. Serum HGF levels are mainly connected with liver fibrosis and elevated levels of transforming growth factor beta 1 (TGF‐β1).^96^ The HGF/c‐Met pathway could offer intriguing inhibitors for HCV.^97^ Because there is also HGF‐activated CCAAT/enhancer‐binding protein‐β (C/EBPβ), which binds to receptor c‐Met to induce hepatocyte survival upon phosphorylation.^26^ The related mechanisms in viral diseases are presented in Figure 4.

### 3.2 COVID‐19

The virus that is responsible for severe acute respiratory syndrome coronavirus 2 (SARS‐CoV‐2) causes COVID‐19, a transmissible illness that is one of the most common illnesses to affect human health in recent years. When an infection occurs, the body's defense mechanism produces cytokines called pro‐inflammatory cytokines, which motivate the stromal cell population to produce pro‐HGF, or hepatocyte growth element, which is then activated as HGF.^93^, ^98^ As a pleiotropic cytokine with anti‐inflammatory properties, HGF plays a critical role in the repair of injured lung tissue,^96^ participates in the regulation of coagulation in vascular and endothelial cells,^92^ and serves as a prognostic biomarker as well as a reliable predictor of mortality and poor clinical outcomes in affected patients.^96^ Notably, sustained upregulation of HGF has been observed in COVID‐19 patients, and dysregulation of apoptotic signaling represents one of the key pathological features of the disease^99^; while endothelial injury and aberrant angiogenesis are hallmarks of neovascularization and pulmonary fibrosis in COVID‐19, these processes have also been reported to promote cell migration via HGF‐dependent signaling.^96^, ^100^ In terms of therapeutic potential, mesenchymal stem cell (MSC)‐based therapy exerts immunomodulatory effects partly through the production of HGF, and binding of HGF to its receptor tyrosine–protein kinase Met (c‐Met) activates downstream signaling that attenuates COVID‐19‐associated pulmonary fibrosis and improves lung function via paracrine mechanisms.^38^, ^100^ In summary, lower cycle threshold (Ct) values from polymerase chain reaction in the early phase of infection correlate with higher viral activity, whereas elevated antibody responses are associated with increased disease severity,^39^ and host‐directed immunotherapies aiming to counteract hyperactivated pro‐inflammatory immune responses have emerged as a promising direction—targeting the HGF/c‐Met signaling pathway, in particular, may therefore represent a novel and effective therapeutic strategy for the management of COVID‐19.^96^

### 3.3 HGF/c‐Met pathway in viral pathologies

Alveolar epithelial cells represent the primary cellular targets of influenza virus infection. Previous studies have established that signaling cascades including transforming growth factor α/epidermal growth factor receptor and HGF/c‐Met modulate the repair of injured bronchial epithelium by facilitating cellular migration and proliferation.^99^, ^101^ Beyond influenza, Kaposi's sarcoma‐associated herpesvirus (KSHV)—a key etiological agent of primary effusion lymphoma (PEL)—markedly induces activation of the HGF/c‐Met pathway in both in vitro and in vivo experimental systems; conversely, pharmacological or genetic inhibition of this signaling axis triggers cell cycle arrest, DNA damage, and apoptotic cell death in PEL‐derived cells, underscoring its pathogenic relevance in viral oncogenesis.^102^, ^103^ Notably, patients with active systemic viral infections (encompassing influenza, KSHV, and other viral pathogens) consistently display significantly elevated serum concentrations of HGF and epidermal growth factor, suggesting these cytokines may serve as general biomarkers of viral‐mediated immune dysregulation. In the context of hepatitis B virus (HBV)—the leading cause of chronic liver disease and progressive hepatic cirrhosis globally.^104^, ^105^ Tumors exhibit susceptibility to small molecule Met inhibitors when HGF triggers autocrine activation, suggesting that HGF is a driver of HBV‐induced HCC development and could be a useful biomarker for Met‐targeted therapy.^106^

## 4 HGF/c‐MET IN DAMAGE REPAIR

### 4.1 Kidney injury

**FIGURE 5:**
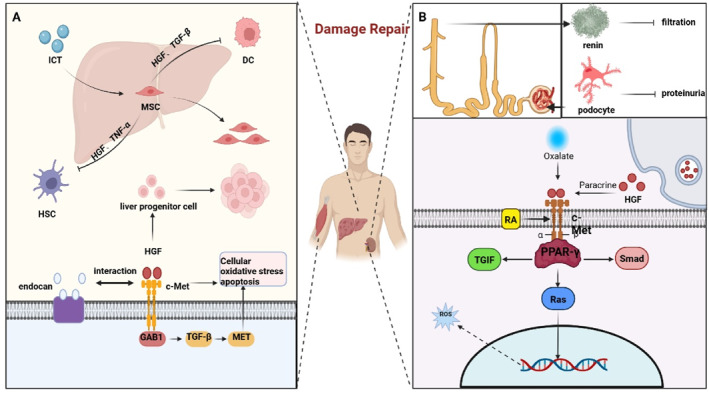
HGF/c‐Met in damage repair. (A) ICT stimulates the value‐added of MSC to promote the secretion of HGF, which affects its production of exosomes influencing the value‐added effect on the cells, and Endocan interacts with HGF/c‐Met to affect the hepatocyte population, and at the same time it can control the TGF‐β thus affecting its hepatic repair of the damage. (B) HGF prevents apoptosis of renal tubular cells while podocytes prevent proteinuria, whereas the HGF/c‐Met pathway is essential for activation of podocytes after injury, whereas oxalate induces intracellular ROS produces antagonistic PPAR‐γ, which controls the activation of SAMD‐containing and regulates the course of the whole pathway. HGF, hepatocyte growth factor; ICT, icariin; MSC, mesenchymal stem cell; PPAR‐γ, peroxisome proliferator‐activated receptor gramma; ROS, reactive oxygen species; SAMD, sterile alpha motif domain; TGF‐β, transforming growth factor beta.

HGF is substantially expressed in rats with glycerol‐induced severe renal damage (Gly ARF) and functions to prevent renal tubular cell apoptosis.^107^ Administration of interleukin‐6 (IL‐6) to Gly ARF rats further upregulates c‐Met expression, which activates the HGF/c‐Met signaling pathway and promotes kidney repair.^40^, ^107^, ^108^ Nevertheless, excessive activation of the HGF/c‐Met pathway may induce nephrotoxicity and contribute to the development of partial drug resistance.^40^ Nephrin, a protein expressed between podocytes, enables podocytes to act as a glomerular filtration barrier and prevent proteinuria.^40^ Activation of the HGF/c‐Met pathway is essential for podocyte survival after injury; additionally, it increases the expression of synaptopodin and adenosine in podocytes.^41^, ^109^ Oxalate antagonizes peroxisome proliferator‐activated receptor gramma (PPAR‐γ) and induces intracellular reactive oxygen species production. As an upstream regulator of the HGF/c‐Met pathway, PPAR‐γ can modulate the expression of mothers against decapentaplegic 7 (Smad7), which inactivates the TGF‐β1/Smad signaling pathway while activating the HGF/c‐Met signaling pathway.^41^ Accordingly, rosiglitazone (RSG), a PPAR‐γ agonist, can be used for the treatment of kidney stones via this mechanism.^109^ In mesangial cells, 9‐cis retinoic acid (RA) activates the HGF/c‐Met pathway, which in turn induces the production of TG‐interacting factor (TGIF)—a Smad transcriptional co‐repressor. Thus, RA may enhance kidney damage repair and reverse the antifibrotic effects of mesangial cells.^42^ The protective roles in tissue injury repair are clearly illustrated in Figure 5.

### 4.2 Liver injury

Acute liver failure (ALF) is a life‐threatening condition, but patients may achieve recovery with MSCs‐based therapy.^110^ The HGF/c‐Met signaling pathway is closely associated with liver injury repair, as HGF and transforming growth factor β (TGF‐β) are key signaling molecules for liver regeneration. Icariin (ICT), a bioactive flavonoid, can promote MSC proliferation and enhance their anti‐apoptotic capacity by activating the HGF/c‐Met pathway, thereby holding therapeutic potential for ALF.^110^, ^111^, ^112^ Dioscin (a natural steroidal saponin extracted from traditional Chinese medicinal plants such as *Dioscorea bulbifera* L.) modulates the expression of ICAM‐1, vimentin, HGF/c‐Met, and GSTA1 to suppress cell apoptosis and lipid peroxidation; it exerts a therapeutic effect on chemical‐induced acute liver injury by suppressing cell apoptosis, necrosis, inflammatory cytokine release, and lipid peroxidation.^113^ Endocan (a secreted heparan sulfate proteoglycan involved in angiogenesis and stem cell regulation) modulates the proliferation of hepatic stem cells by interacting with the HGF/c‐Met signaling pathway.^43^, ^114^ Following hepatectomy, DEP domain‐containing mTOR‐interacting protein (Deptor) modulates the HGF/c‐Met axis to enhance hepatocyte proliferation and reduce hepatocyte susceptibility to necrosis.^43^, ^115^ A study by Li et al. demonstrated that the HGF/c‐Met pathway can regulate the TGF‐β‐mediated EMT response, thereby controlling hepatocyte apoptosis and oxidative stress while enhancing the regenerative potential of hepatic progenitor cells.^112^ In non‐alcoholic steatohepatitis, activation of the HGF/c‐Met pathway promotes Janus kinase 2‐signal transducer and activator of transcription 3 (Jak2‐STAT3) phosphorylation, suppresses inflammation, and improves liver function.^111^, ^112^ Collectively, a variety of therapeutic strategies targeting the HGF/c‐Met pathway have been shown to promote liver injury repair, providing a foundation for further research into liver disease treatments.

### 4.3 Muscle injury

Muscle regeneration activates the HGF/c‐Met pathway. Among the different infiltrating cell types, the macrophage is the most affected. The HGF/c‐Met pathway regulates the M1 to M2 macrophage polarization transition, whereas the AMP‐activated protein kinase/calcium/calmodulin‐dependent protein kinase beta signaling pathway also modulates M2 macrophage polarization—together promoting muscle regeneration after injury.^116^, ^117^ Furthermore, HGF acts in an autocrine manner on macrophages in damaged muscles.^112^ Acute myocardial infarction (AMI) impairs patients' health and reduces their quality of life, and the prognosis of AMI patients with diabetes mellitus (DM) is poorer.^118^ Notably, damaged cardiac cells exhibit increased expression of HGF and c‐Met at both mRNA and protein levels; HGF can further enhance HGF/c‐Met signaling, thereby playing a crucial role in inhibiting myocardial apoptosis and improving cardiac function in DM‐associated AMI.^118^ MSC have the capacity to regenerate damaged tissues: They can initiate chemoattraction by triggering apoptosis via the HGF/c‐Met axis.^118^ Therefore, the cooperation between apoptosis inhibition (in cardiac cells) and apoptosis‐mediated MSC chemoattraction enables the regeneration and recovery of damaged cardiac cells.^112^, ^119^

### 4.4 HGF/c‐Met in tissue injury and regeneration

In the central nervous system CNS, HGF has trophic effects.^120^ In astrocytes of rats with spinal cord injury, HGF and c‐Met expression is elevated during both the subacute and chronic phases; thus, the HGF/c‐Met pathway may be activated following trauma and associated with reparative regeneration.^9^, ^121^ In reserpine‐induced gastric lesions, HGF/c‐Met expression is increased, whereas amylin can specifically protect the gastric mucosa and regulate HGF/c‐Met expression.^121^ MSCs secrete various paracrine factors to facilitate damage repair. A study by Xu et al. demonstrated that MSCs transfected with the HGF gene exert a more favorable effect on endometrial regeneration.^44^, ^122^

## 5 HGF/c‐MET SIGNALING IN DIABETES AND NEURODEGENERATIVE DISORDERS

### 5.1 Diabetes

Diabetes is a common metabolic disease that can lead to many serious side effects, including kidney and reproductive impairment.^123^ It also affects the function of various pancreatic cells. Previous studies have clearly demonstrated that HGF/c‐Met signaling is activated in diabetic rats, and this pathway is closely associated with pancreatic cell function.^124^ During pancreatic development, neogenesis, and regeneration, pancreatic stem cells (PSCs) give rise to endocrine, acinar, and ductal cells; the HGF/c‐Met pathway is critical for the regeneration of the adult pancreas from stem/progenitor cells and is also essential for pancreatic development.^125^ Notably, this pathway may help delay β‐cell death in diabetic patients: nuclear factor‐κB (NF‐κB) is involved in cell death, and increased NF‐κB activation leads to elevated nitric oxide (NO) production and enhanced β‐cell susceptibility to damage. However, the HGF/c‐Met pathway may inhibit the NF‐κB‐inducible NO synthase (iNOS)‐NO pathway in β‐cells to protect these cells.^126^

Diabetes is classified into two main subtypes: type 1 and type 2 diabetes.^127^ Type 2 diabetes is associated with insulin resistance and β‐cell dysfunction. In type 2 diabetes, the HGF/c‐Met pathway and its downstream signaling pathways may improve β‐cell function.^128^ In type 1 diabetes as well, β‐cell regeneration requires HGF/c‐Met activation.^129^ Gestational diabetes mellitus may be associated with impairment of the HGF/c‐Met pathway: Adipocyte growth relies on both growth differentiation factor 15 and HGF, and this growth is linked to the overexpression of forkhead box M1 (FoxM1) and downregulation of p12. HGF may downregulate p12 in β‐cells, though it remains unclear whether HGF regulates FoxM1 in β‐cells.^130^ Diabetes‐related problems are associated with oxidative stress. The HGF/c‐Met pathway is known to contribute to oxidative stress. It is unknown, therefore, if the two factors have an impact on testicular damage brought on by diabetes.^123^ The HGF/c‐Met pathway may be targeted collectively for the treatment of diabetes.

### 5.2 Alzheimer's disease

Alzheimer's disease (AD) is a neurodegenerative disorder with a complex pathophysiology involving neuroinflammation, protein aggregation, and other factors.^131^ Emerging evidence indicates that the HGF/c‐Met pathway exerts both neurotrophic and neuroprotective effects: Its activation can promote neurogenesis, neuronal survival, synaptogenesis, and synaptic plasticity, thereby supporting its potential as a neurotrophic factor‐based therapeutic strategy for AD.^132^, ^133^ Parkinson's disease (PD) is another neurodegenerative disorder with an increasing incidence, characterized by the loss of dopaminergic (DA) neurons.^134^ The HGF/c‐Met pathway may protect DA neurotransmission; however, direct administration of HGF is not a viable solution. Consequently, researchers have identified a small‐molecule HGF mimetic with improved efficacy compared to previous candidates, though its clinical availability and potential for long‐term treatment remain to be established.^42^, ^135^ Collectively, therapeutic targeting of the HGF/c‐Met pathway holds promise for both AD and PD.

## 6 CONCLUSIONS

This review systematically summarizes the multifaceted roles of the HGF/c‐Met pathway in the pathogenesis of diverse diseases and its potential as a therapeutic target. Accumulating evidence confirms that the HGF/c‐Met pathway is not only a key driver of malignant progression in multiple malignancies—including lung cancer, colorectal cancer, PC, and renal cell carcinoma—but also a critical mediator of treatment resistance in these tumors; diosgenin, monoclonal antibody 3936, and miR‐200a overexpression block HGF‐mediated pathway activation; whereas 5‐FU, BFE, and CTX III represent dual‐targeting agents that simultaneously inhibit HGF and c‐Met. Collectively, these strategies achieve anti‐tumor efficacy by abrogating the pro‐malignant functions of the HGF/c‐Met pathway.

Beyond oncology, the HGF/c‐Met pathway plays a pivotal role in tissue repair and regeneration following injury to organs such as the kidney, liver, muscle, and heart. For instance, RSG and 9‐cis RA activate this pathway via distinct mechanisms to mitigate renal injury, though excessive activation may paradoxically induce nephrotoxicity and drug resistance. ICT and diosgenin promote liver repair by enhancing HGF/c‐Met signaling, whereas pathway activation facilitates muscle regeneration post‐injury and regulates cardiomyocyte apoptosis to protect cardiac function. In metabolic and neurodegenerative diseases, the HGF/c‐Met pathway also exerts protective effects: It safeguards pancreatic β‐cells by downregulating p12 expression and inhibiting the NF‐κB‐iNOS‐NO axis, creating a favorable microenvironment for β‐cell survival and function in diabetes, additionally, its neurotrophic and neuroprotective properties render it a promising therapeutic candidate for AD and PD.

Notably, the therapeutic modulation of the HGF/c‐Met pathway is context‐dependent: Aberrant activation must be suppressed to curb tumor progression, whereas controlled activation is beneficial for tissue repair, metabolic homeostasis, and neuroprotection. Despite significant progress, several critical gaps remain: The molecular mechanisms underlying pathway crosstalk with other oncogenic or reparative signaling networks (e.g., PI3K/AKT or TGF‐β) are not fully elucidated; the development of resistance to HGF/c‐Met‐targeted agents remains a major clinical challenge; and the lack of reliable predictive biomarkers hinders the stratification of patients most likely to benefit from such therapies. Future research should focus on deciphering these pathway interactions, identifying novel resistance mechanisms, and developing highly selective, tissue‐specific modulators of the HGF/c‐Met pathway. Moreover, clinical trials combining HGF/c‐Met inhibitors with other therapeutic modalities (e.g., immunotherapy or chemotherapy) hold promise for improving treatment outcomes in cancer, whereas targeted activation strategies may offer new avenues for managing tissue injury, diabetes, and neurodegenerative diseases.

In conclusion, the HGF/c‐Met pathway serves as a double‐edged sword in human health, exerting pro‐malignant effects in tumors yet protective functions in tissue repair and chronic diseases. Precise context‐specific modulation of this pathway, guided by a deeper understanding of its molecular mechanisms and clinical relevance, will be crucial for translating preclinical findings into effective therapeutic strategies across a broad spectrum of diseases.

## 7 FUTURE PERSPECTIVE

Given that the HGF/c‐Met axis mediates a wide spectrum of biological responses across diverse tissues, targeted modulation of this signaling cascade holds substantial translational potential for clinical application. The HGF/c‐Met pathway has attracted increasing attention as a candidate target for prognosis and intervention in tissue injury repair, cancer therapy, diabetes management, and other pathological conditions. A critical prerequisite for clinical translation is the identification and validation of reliable biomarkers to reflect pathway activity. Key indicators in tissue repair include HGF, c‐Met, phospho‐c‐Met, PPAR‐γ, and Smad7, whose expression can be quantitatively evaluated by immunohistochemistry. Such detection has confirmed the involvement of the HGF/c‐Met pathway in renal, muscular, and hepatic injury repair.

Several candidate agents have shown promise in treating tissue and organ damage. For instance, ICT upregulates HGF/c‐Met signaling in ALF and facilitates hepatic recovery; however, the precise mechanism by which ICT prevents stem cell apoptosis via the HGF/c‐Met pathway remains incompletely understood.^110^ Similarly, HGF/c‐Met signaling protects podocytes in renal injury models, yet the in vivo effects of exogenous human HGF administration remain unclear due to the lack of standardized delivery systems in preclinical studies.^109^

In malignant diseases, established biomarkers of the HGF/c‐Met axis include HGF, c‐Met, PI3Kα, and miR‐200a. Although tyrosine kinase inhibitors (TKIs) represent conventional targeted agents, many non‐selectively inhibit multiple tyrosine kinase domains rather than specifically targeting c‐Met, leading to off‐target effects and eventual drug resistance. Consequently, the limited efficacy of current TKIs underscores the demand for more precise therapeutic strategies.^47^, ^48^ Notably, DFX117 exerts enhanced anti‐tumor activity in lung cancer by simultaneously inhibiting c‐Met and PI3K,^46^ whereas miR‐200a enables gene‐level suppression of the HGF/c‐Met pathway, thus achieving more specific therapeutic effects.^47^

In diabetes, molecules including HGF, c‐Met, miR‐101‐3p, p65/NF‐κB, Akt, GSK‐3, and p27 are closely associated with disease onset, progression, and therapeutic response. Evaluating the interactions and functional relevance of these factors is essential for optimizing diabetes treatment. Nonetheless, several key questions remain unresolved. For example, administration of HGF or other growth factors to promote regeneration may induce unintended proliferation of non‐target cell populations beyond pancreatic β‐cells.^129^ Furthermore, the direct contribution of HGF and the HGF/c‐Met cascade to adaptive β‐cell hyperfunction during pregnancy remains to be clarified.^126^

Finally, given the divergent roles of the HGF/c‐Met pathway in different diseases—being pathogenic in cancer but regenerative in tissue repair—treatment strategies must be tailored accordingly: inhibition in malignancies versus activation in injury recovery. Single‐targeting of either HGF or c‐Met alone may lead to off‐target consequences and incomplete responses. Emerging evidence suggests that combinatorial targeting, guided by immunohistochemically validated biomarkers, may minimize such limitations and improve therapeutic precision. Accordingly, context‐dependent biomarker‐guided modulation of the HGF/c‐Met axis represents a more rational and effective approach for future disease management.

## AUTHOR CONTRIBUTIONS

Hongqin Sun and Hao Shu performed the data analysis and interpreted the data, Qian Zhang, Yunyan Ma, and Jishan Zheng prepared the draft. Dengwang Chen and Jidong Zhang revised the draft. Tao Song designed the research and supervised all the work. All authors contributed to the article and approved the submitted version.

## CONFLICT OF INTEREST STATEMENT

The authors declare no conflicts of interest.

## ETHICS STATEMENT

Because the current study is a review based on published literatures, it does not involve any human participants, human tissues, or animal experiments. Ethical approval is not applicable to this article.

## Data Availability

The datasets generated during and/or analyzed during the current study are available from the corresponding author on reasonable request. (Note: As this is a review, the data refer to the literatures cited.)
